# An update on the Symbiotic Genomes Database (SymGenDB): a collection of metadata, genomic, genetic and protein sequences, orthologs and metabolic networks of symbiotic organisms

**DOI:** 10.1093/database/baz160

**Published:** 2020-02-13

**Authors:** Mariana Reyes-Prieto, Carlos Vargas-Chávez, Mercè Llabrés, Pere Palmer, Amparo Latorre, Andrés Moya

**Affiliations:** 1 Evolutionary Systems Biology of Symbionts, Institute for Integrative Systems Biology (I^2^SysBio), Universitat de València, Paterna, València, Spain; 2 Sequencing and Bioinformatics Service, Foundation for the Promotion of Sanitary and Biomedical Research of the Valencia Region (FISABIO), València, Spain; 3 Functional and Evolutionary Genomics, Institute of Evolutionary Biology (IBE), CSIC-Universitat Pompeu Fabra, Barcelona, Spain; 4 Department of Mathematics and Computer Science, University of the Balearic Islands, Palma, Balearic Islands, Spain; 5 Genomic and Health Area, Foundation for the Promotion of Sanitary and Biomedical Research of the Valencia Region (FISABIO), València, Spain; 6 CIBER in Epidemiology and Public Health (CIBEResp), Madrid, Spain

## Abstract

The Symbiotic Genomes Database (SymGenDB; http://symbiogenomesdb.uv.es/) is a public resource of manually curated associations between organisms involved in symbiotic relationships, maintaining a catalog of completely sequenced/finished bacterial genomes exclusively. It originally consisted of three modules where users could search for the bacteria involved in a specific symbiotic relationship, their genomes and their genes (including their orthologs). In this update, we present an additional module that includes a representation of the metabolic network of each organism included in the database, as Directed Acyclic Graphs (MetaDAGs). This module provides unique opportunities to explore the metabolism of each individual organism and/or to evaluate the shared and joint metabolic capabilities of the organisms of the same genera included in our listing, allowing users to construct predictive analyses of metabolic associations and complementation between systems. We also report a ~25% increase in manually curated content in the database, i.e. bacterial genomes and their associations, with a final count of 2328 bacterial genomes associated to 498 hosts. We describe new querying possibilities for all the modules, as well as new display features for the MetaDAGs module, providing a relevant range of content and utility. This update continues to improve SymGenDB and can help elucidate the mechanisms by which organisms depend on each other.

## Introduction

Symbiotic relationships between bacteria and eukaryotes occur naturally and ubiquitously in nature. It is a general principle in the evolution of eukaryotes, and an important selective force behind evolution. The correct functioning of all ecosystems depends on these interactions (1–4). To our knowledge, this is the first database completely devoted to symbiotic interactions. The Symbiotic Genomes Database (SymGenDB, previously named SymbioticGenomesDB; http://symbiogenomesdb.uv.es/) is a public resource that provides information on symbiotic relationships throughout the tree of life (5). We use bioinformatic tools and manual curation to create a comprehensive list of associations between symbiotic organisms, and match them to their metadata, their genomic and genetic content, their association to orthologous genes and their metabolic networks in a novel format known as Directed Acyclic Graphs (MetaDAGs) (6). SymGenDB focuses on accessibility, so we use ids from the primary databases, NCBI and KEGG (7–10), for taxonomy, organism’s names and gene ids, so the accessibility to information is as complete as possible and enables data to be compared across species/strains, among other advantages. In addition, we have included links to the same primary databases in the modules of SymGenDB, as well as links to each organism’s scientific literature. This process of compiling a comprehensive list with community-accepted controlled ids and accession identifiers ensures that the content of SymGenDB is cohesive, controllable and computable, and also complying to the FAIR principle (Findable, Accessible, Interoperable and Reusable) (11).

In this work, we provide the first SymGenDB update and describe our newly released module that focuses on the metabolism of each organism included in the database. We also comment on the advanced query searches we have implemented and the new tools that allow us to visualize metabolic interactions in a new manner. Researchers in the world of symbiosis and other fields (microbiome, for example), can benefit from SymGenDB to explore associations between organisms and quickly generate data and testable hypotheses about the molecular mechanisms beneath these types of relationships.

## Database contents—an overview

SymGenDB consists of four modules that serve different purposes. The first three, organisms, genomes and genes, have been previously described in the first publication of this database (5). Although a small overview is listed in the next segments for each module, we invite users to refer to the original paper for detailed functionality, and to browse the database and watch the videos of the quick tour we offer.

This update maintains the same general architecture of the previous version of the database, although the scripts required major modifications to adapt to the new data source. While the previous version relied on MGDB (12), this version uses KEGG as the single source of data. The raw files from KEGG were parsed using custom scripts written in R to format the data and organize it in efficient objects that allow quickly retrieving the desired information.

## Module organisms

**Figure 1:**
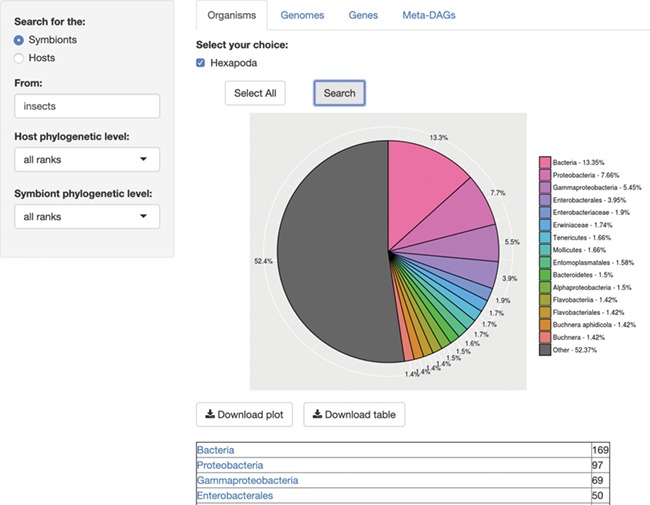
SymGenDB’s Organisms module. For this example, we searched for the symbionts of insects included in SymGenDB. It is worth noting that we used the term ‘insects’ and the output shows the scientific name ‘Hexapoda’ because the database includes a hefty list of synonyms so users do not have to know/search only for scientific names. We searched for both the host and the symbiont’s phylogenetic level as default, and the result shows an abundance chart (only the first 15 most abundant hits are displayed in color, the rest are encompassed in grey) and a list of organisms showing all the hits. Each resulting ‘organism’ (or family, genus, class, etc.) is a link to its NCBI taxonomy page. The resulting list shown in this figure is cut short for spacing purposes.

First, we encounter the module ‘Organisms’, where users can get an overview on an/the organism(s) involved in a symbiotic relationship. One of the best features of this database is that users can search for either a host or a symbiont, at any taxonomic levels, where the default = all taxonomic levels, as ‘all ranks’ (in accordance with NCBI (13, 14)). The output of this search consists of a chart containing the relative abundance of the organisms associated to the searched query, and a list of organisms by taxonomy level resulting from the search, with links to the Taxonomy Browser of NCBI (Figure 1). The chart and the list are both available for download.

**Figure 2:**
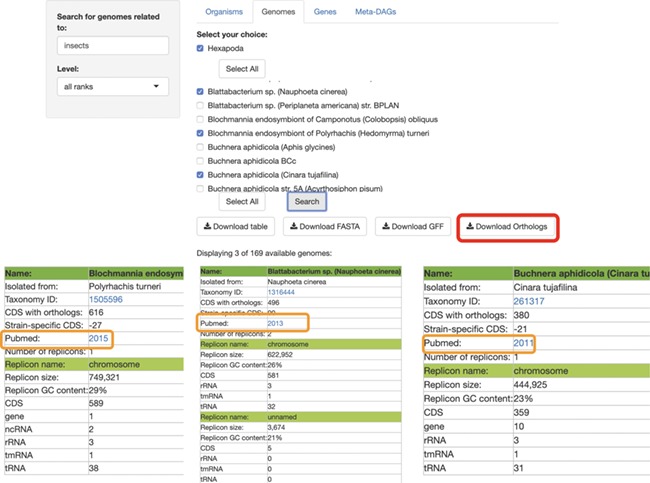
SymGenDB’s Genomes module. Continuing with the example on symbionts of insects, we searched for those genomes and selected 3 of the 169 available in SymGenDB’s. The resulting table (viewed horizontally for spacing purposes, although it is presented vertically in the database), consists of all the metadata of the genomes of our choice, with a link to the host’s taxonomy id from NCBI. It is important to show that one of the downloads available in this module is the orthology table of the chosen genomes (shown in red) which is very helpful for evolutionary research. Furthermore, the literature where this genome was first described is also made available to users (shown in orange).

## Module genomes

In the next module, ‘Genomes’, searches retrieve all the metadata included in the database for each resulting symbiont: Name (the complete name of the bacteria), Host (described as the isolation source, to avoid complications regarding host diversity), Taxonomy ID (NCBI’s ID), the number of CDS with orthologs, the Strain-specific CDS, the number of replicons within this genome and a link to the organism’s scientific literature (the first paper were the symbiotic relationship was described). For each replicon in the genome, we also provide information of the replicon name, its size, GC content, CDSs, genes, ncRNAs, rRNAs, tmRNAs and tRNAs. The search can also be performed at all different taxonomic levels, with the default = all taxonomic levels as ‘all ranks’ (Figure 2). Moreover, a table with all the metadata of the genomes included in the results, a fasta file with all genomes, a gff file of all genomes and the orthologs shared by all these genomes are available for download in this module.

## Module genes

**Figure 3:**
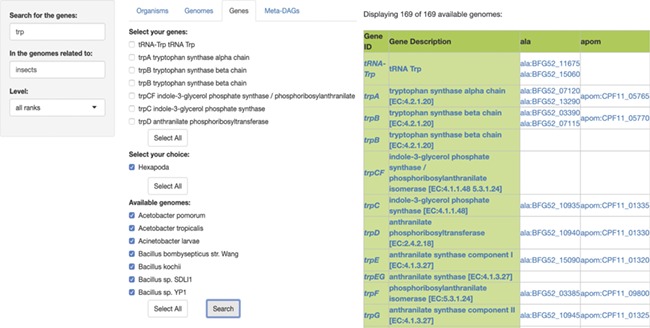
SymGen’s gene module. For this example, we searched for all the genes containing the letters ‘trp’ in the genomes of symbionts of insects. We selected all of the 169 available genomes. The resulting table is a list in a presence/absence format, where all the present genes are shown with their KEGG id and a link to their KEGG gene web page. The ‘Gene ID’ and the ‘Gene Description’ features are both linked to their KEGG orthology web page.

In the third module, ‘Genes’, users are able to search for specific genes in the genomes of symbiotic organisms. The search involves the input of two queries, first the name of the gene(s) to search for and then, the name of the symbiotic organisms to search for, or the name of the host of the symbiotic organism to search for. The output is a presence/absence list of genes with their KEGG id and description, in every genome available as a result. Each resulting gene is also linked to its KEGG gene web page (Figure 3). The ‘Gene ID’ and the ‘Gene Description’ features are both linked to the KEGG ortholog web page of each result. Both the gene table and the amino acid sequences of the results are downloadable.

A couple of remarks to keep in mind while searching in all the modules at SymGenDB are as follows: the first one is that all synonyms of NCBI are linked to the organisms’ names to facilitate searches. For example, if a user wants to search for the symbionts associated to human, he/she can write the words ‘man’ or ‘human’ or common misspellings such as ‘Homo sapience’ and still get the output for *Homo sapiens*, its correct taxonomic name. Also, searches of more than one organism/genome/gene can be made at the same time. The only requirement is that queries must be separated by a comma (‘,’).

## Increased data content

One of the most important aspects of databases in the genomic era is their maintenance and updates. SymGenDB became publicly available in 2015, and two updates to the data content of the database have been made since then. As of July 2019, SymGenDB contains 2328 symbiotic genomes, associated to 498 hosts (Table 1), presenting a roughly 25% increase from our previous version. The interactions between symbionts and hosts are manually curated from a cross reference of the lists of organisms included in KEGG and the JGI’s GOLD Genomes Online Database (15). These associations are mainly revised in peer-reviewed scientific papers from PubMed. Next, all the metadata and genomic data associated to a symbiont in the list is retrieved from the KEGG archives.

**Table 1:** Update on the data content of SymGenDB

Data	Previous version	Update July 2019
Symbiotic genomes	1955	2328
Hosts	384	498
Orthologous genes	3 121 262	3 808 086
Orthologous clusters	7567	8478

## New module—MetaDAGs

We have added a new module to SymGenDB consisting of the metabolic networks of all organisms modeled as directed acyclic graphs (MetaDAGs). The MetaDAG of every organism is obtained as a suitable reduction of the reaction graph created with the metabolic data of each genome we retrieved from KEGG. In the reaction graph model, the nodes are the reactions and there is an arc (a directed edge) between two reactions if some metabolite in the product of the source-arc reaction is in the substrate of the target-arc reaction. Next, the strongly connected components in the reaction graph are collapsed into a single node, called a metabolic building block. The result is a graph with no cycles, hence a directed acyclic graph called a MetaDAG. The MetaDAG keeps the connectivity of the metabolic network but reduces considerably the number of nodes, which facilitates its visualization. Notice that the nodes in the MetaDAG are the strongly connected components in the reaction graph, which consist of one or many reactions. In order to easily visualize the size of every metabolic building block of the MetaDAG, we scale the size of the corresponding node depending on the number of reactions included in the node, and use two colors to distinguish these: green when the metabolic building block has only one reaction, and yellow when there are two or more reactions. To contextualize the metabolic building blocks, we present an interactive metaDAG where users are capable to scroll over each node to visualize the reactions it contains, as well as to highlight their position in the global metabolic pathway’s map from KEGG. Furthermore, we distinguish cut nodes (those that, if removed, they disconnect the network), by drawing an octagon instead of a circle for the node. The methodology for these calculations is described in detail in Alberich et al. 2017 (6).

**Figure 4:**
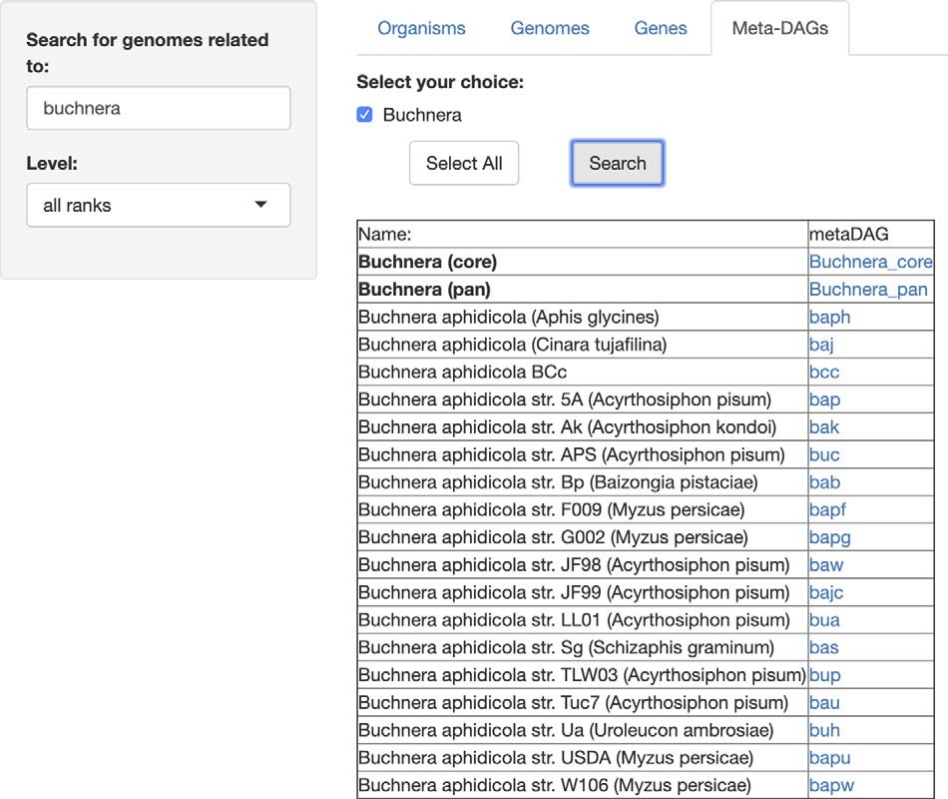
SymGenDB’s new MetaDAGs module. In this example, we search for the symbionts of the genus ‘Buchnera’. The output is a list of organisms as bacterial strains, as well as the joint (pan) or intersecting (core) metabolism of the strains resulted in the search, included in the taxonomic level ‘genus’ (in bold). It is important to denote that in the case of the ‘pan’ and ‘core’ interacting metabolism, not only the genomes of the bacterial strains resulting from the search are presented. The complete set of strains of the same genus available in SymGenDB constitutes these MetaDAGs.

For this last module, ‘MetaDAGs’, users are able to search for the genome-scale metabolic network as MetaDAGs, related to any symbiont (or first, search for the host and then select the symbiotic genomes associated to that host). As a running example, we present the search for the symbionts included in SymGenDB of the genus *Buchnera*, known symbionts of aphids (Figure 4). The result is a list of the bacterial strains included in the symbiotic relationship the user searched for, associated to a link. When the link of interest is clicked on, a new visualizer shows its MetaDAG in a dynamic manner, where the user can zoom in and out of any part of the graph, and click on any node to get a couple of pop-up windows. The first window has the information on the reaction(s) included in that node. In this pop-up window, users can also click on the image of each reaction to see it bigger and better and, by clicking on the link ‘more info’, get to KEGG’s reaction web page for that reaction. We have also included the information of other organisms that present the same reaction (by clicking on the link ‘graphs’ users get a full display), which can be very useful for orthology and evolutionary studies, among others. The second window shows the global metabolic pathways map from the KEGG’s web page with the reactions of the metabolic building blocks highlighted. This implementation contextualizes the metabolic building blocks to the well-known global metabolic description map.

Furthermore, in the first result after searching for the MetaDAGs of interest, we also offer the core MetaDAG and the pan MetaDAG of the bacterial strains of the same genus. The core MetaDAG is obtained by considering the common metabolic building blocks present in all the MetaDAGs of the bacterial strains of the same genus. That is, the nodes (i.e. metabolic building blocks) in the core MetaDAG are the intersection of the nodes of the MetaDAGs of the bacterial strains of the same genus with the corresponding directed acyclic graph topology. On the other hand, the pan MetaDAG is obtained by considering the union of the nodes (metabolic building blocks) in all the MetaDAGs of the bacterial strains of the same genus with the corresponding directed acyclic graph topology. It is important to inform that in the case of the ‘pan’ and ‘core’ interacting metabolism, in certain cases where all the organisms of the same genus are not part of the symbiotic relationship searched for, not only the genomes of the bacterial strains resulting of the search are presented. The complete set of strains of the same genus available in SymGenDB constitutes these pan and core MetaDAGs.

**Figure 5:**
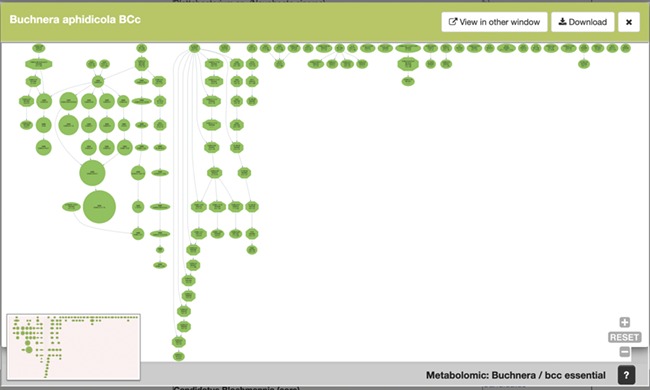
SymGenDB’s new MetaDAGs module’s output. In this example, we search for the symbionts of the genus ‘Buchnera’. The output is a preview of a graphical display of the MetaDAG you get by choosing Buchnera aphidicola from *Cinara tujafilina*. This dynamic display can be viewed in another window, downloaded as a PDF and all the information of the reaction(s) included in each node(s) is available by clicking on the node(s) of interest.

To continue with our example, Figure 5 shows an example of the graphical output of our MetaDAGs module. From the list of strains resulting from the search, we clicked on *Buchnera aphidicola* from *Cinara tujafilina*, and a preview of a graphical display of the MetaDAG is shown. This display can be viewed in another window (most MetaDAGs are big and space is needed to fully appreciate the graph, as well as to get into details), download the graph as a PDF and/or dismiss the preview.

**Figure 6:**
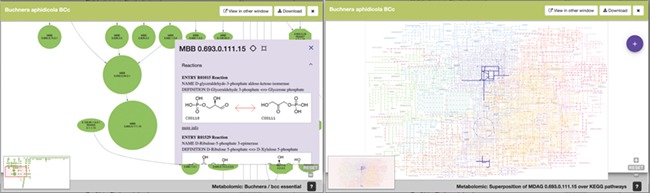
SymGenDB’s new MetaDAGs module’s output where users can not only see the reactions included in their graph of interest in detail but also contextualize the reaction within the KEGG metabolic map. We have included a feature to highlight the reaction(s) of interest for this purpose.

Lastly, Figure 6 is an example of the visualization of the pop-up windows that emerge from one node of the metaDAG of *B. aphidicola* from *C. tujafilina.*

## Availability of web interface and services

The web interface of SymGenDB has been modified from its previous version. We have included different tabs to make the interface and services of the database more understandable:

- Home, where an overview of the purpose of the database, as well as its metrics and a phylogenetic tree including all the bacterial strains included in our catalogue is shown (http://symbiogenomesdb.uv.es/).
- Database, where in turn, all the different modules of the database are shown in tabs. This is where all the searches and results are presented (http://symbiogenomesdb.uv.es/database.html).
- Functions, in the first paragraph of this tab users can find the basic description of each module of the database and below that, a detailed description of the functions and an overview on how to search in each module and the results to expect (http://symbiogenomesdb.uv.es/functions.html).
- Quick tour, another subset of tabs where in each tab we present a module, a video on how to search for the data in that module and a step-by-step guide on how to search in each module, with pictures for easier interpretation (http://symbiogenomesdb.uv.es/quick-tour.html).
- Citation, Acknowledgements and contact includes the citation for the first article published of the database (5), as well as the funding received for the creation of this project and a form for users to get in touch with the authors where any problem, doubt or suggestion is received and greatly appreciated (http://symbiogenomesdb.uv.es/acknowledgements.html).

## Future directions

It is the author’s intent to continue to develop tools and data for the completion of the database, with new modules and organisms to include as well as updating the database, at least once a year.
